# Facile Synthesis of 20‐nm‐Thick ZIF‐67 Films for Hydrogen Sieving Using β‐Co(OH)_2_ Precursor Nanosheets

**DOI:** 10.1002/anie.202516048

**Published:** 2025-09-21

**Authors:** Xuekui Duan, Yueqing Shen, Kumar Varoon Agrawal

**Affiliations:** ^1^ Laboratory of Advanced Separations École Polytechnique Fédérale de Lausanne Sion 1950 Switzerland

**Keywords:** Gas separation, Membrane, Metal–organic frameworks, Zeolitic imidazolate frameworks, ZIF‐67

## Abstract

Zeolitic imidazolate framework‐67 (ZIF‐67) is an important class of nanoporous materials for high‐performance membranes for gas separation, offering uniform‐sized gas‐selective pores. However, the fabrication of high‐quality sub‐100‐nm‐thick ZIF‐67 membranes, critical for high‐performance separation, has remained elusive. Herein, we report a facile strategy to fabricate ∼20‐nm‐thick ZIF‐67 membranes. We identify a simple and rapid route to prepare ∼1‐nm‐thick β‐Co(OH)_2_ nanosheets as a precursor to ZIF‐67. Highly dispersed β‐Co(OH)_2_ nanosheets are synthesized via a one‐pot reaction at room temperature in just 1 h using ultradilute cobalt and linker concentrations in water. The resulting high‐aspect‐ratio nanosheets yield compact coating on porous polymeric supports. Well‐intergrown ZIF‐67 membranes are prepared by thermal treatment of nanosheets in the presence of a linker and exhibit attractive hydrogen sieving performance at elevated temperatures (up to 250 °C). This approach provides a scalable and versatile platform for hydrogen separation for applications such as precombustion carbon capture.

## Graphical abstract

We report that highly dispersed β‐Co(OH)_2_ nanosheets (∼1‐nm‐thick) could be synthesized via a one‐pot reaction of ultradilute precursors (Co(NO_3_)_2_ + 2‐methyimidazole (HmIm) + H_2_O system) at room temperature in just 1 h. By using thin coatings of the as‐synthesized β‐Co(OH)_2_ nanosheets as self‐sacrificial templates, ∼20‐nm‐thick ZIF‐67 membranes could be fabricated in a facile way. The prepared ZIF‐67 membranes demonstrated attractive H_2_ sieving performance at elevated temperatures (up to 250 °C).

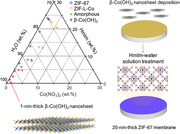

Metal–organic frameworks (MOFs), with their intrinsic nanoporous structure and versatile chemical functionality, represent a key class of nanoporous materials for developing high‐performance molecular sieving membranes.^[^ ^1^, ^2^ ^]^ Zeolitic imidazolate frameworks (ZIFs), which combine zeolite‐like topologies with excellent thermal and chemical stability, represent an important subclass of MOFs for demanding gas separation applications.^[^ ^3^, ^4^ ^]^ Among ZIFs, ZIF‐8 has been the most extensively studied for membrane separations.^[^ ^5^ ^]^ Ultrathin ZIF‐8 membranes with minimal defects are highly desired to maximize membrane performance. This has been attempted by tuning the thin film crystallization process to form well‐intergrown polycrystalline films on porous supports, e.g., enhancing the heterogeneous nucleation (e.g., by increasing the nucleation density)^[^ ^6^, ^7^, ^8^ ^]^ and improving grain intergrowth (i.e., inhibiting Ostwald ripening).^[^ ^9^, ^10^ ^]^

ZIF‐67 is an isostructural analog of ZIF‐8. Both share the same crystal structure and porous topology. They use the same linker (2‐methylimidazole, HmIm). The metal nodes for ZIF‐8 and ZIF‐67 differ and are Zn^2+^ and Co^2+^, respectively. The advantage of ZIF‐67 as a candidate for selective membrane layer is that ZIF‐67 has a stiffer metal‐linker bond than ZIF‐8.^[^ ^11^ ^]^ This makes ZIF‐67 membranes promising for gas separation with increased selectivity. Despite successes in ZIF‐8 membranes, demonstration of an ultrathin ZIF‐67 membrane, needed for high‐permeance separation, has remained limited.

The challenge for obtaining ultrathin ZIF‐67 film is that it crystallizes significantly faster than ZIF‐8 due to the rapid coordination of Co^2+^ ions with HmIm, as compared to Zn^2+^ ions.^[^ ^12^ ^]^ This accelerated crystallization results in fast nucleation and growth, making it challenging to control the formation of ultrathin, defect‐free ZIF‐67 membranes.^[^ ^11^ ^]^

A self‐sacrificial template strategy has emerged as an effective technique to prepare ZIF‐67 membranes. In this method, a sacrificial precursor layer is first deposited on a support. This layer serves as the metal source for conversion into ZIF‐67. Various sacrificial materials have been investigated. For example, Co(OH)_2_ nanosheet network on porous stainless‐steel supports could be successfully converted to ZIF‐67 via hydrothermal^[^ ^13^ ^]^ and solvent‐free ligand treatments.^[^ ^14^ ^]^ Cobalt carbonate hydroxide (CCH) nanowires have been used as the sacrificial layer to prepare ZIF‐67 membranes on porous ceramic tubes.^[^ ^15^ ^]^ Upon further optimization, a more uniform and flat CCH nanolayer could be achieved, which led to compact ZIF‐67 membranes via self‐conversion.^[^ ^16^ ^]^ Co‐based gel films have also been used as a sacrificial layer, which was converted to ZIF‐67 membrane by supercritical fluid processing.^[^ ^17^ ^]^ Co‐MOF nanosheets were utilized as a precursor layer to form ZIF‐67 via the contra‐diffusion method, resulting in honeycomb structures that enhanced gas transport.^[^ ^18^ ^]^

Despite these advancements, most sacrificial layers reported in the literature have considerable thicknesses, leading to ZIF‐67 membranes typically thicker than 1 µm. Although thinner (∼200–500 nm) ZIF‐67 membranes have recently been reported using highly reactive hydroxy salt precursors,^[^ ^19^ ^]^ sub‐100‐nm‐thick ZIF‐67 membranes have not been reported. This is in stark contrast to successful demonstrations of sub‐100‐nm ZIF‐8 membranes, with selective layers as thin as ∼17 nm.^[^ ^4^, ^20^ ^]^ Thus, effective strategies for fabricating ultrathin ZIF‐67 membranes are still lacking.

Herein, we report the synthesis of well‐dispersed, ∼1‐nm‐thick Co(OH)_2_ nanosheets and their application as sacrificial precursors for fabricating ultrathin gas‐sieving ZIF‐67 membranes. The use of these nanosheets enables the formation of uniform, compact coatings on porous polymeric supports. Upon conversion, ZIF‐67 membranes with a thickness of ∼20 nm could be achieved (Scheme 1). These membranes yielded attractive hydrogen‐sieving performance.

**Scheme 1:**
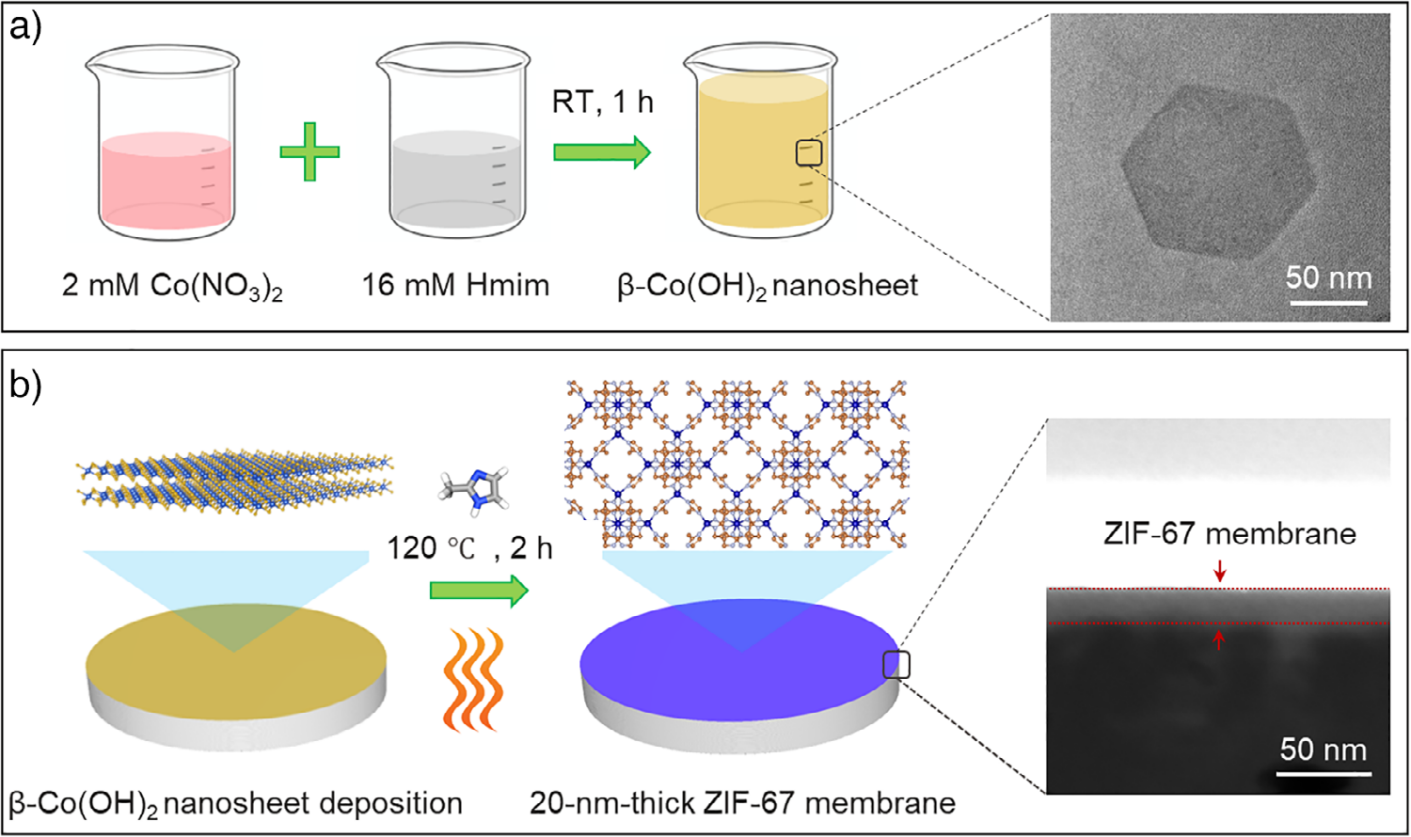
The steps of making ultrathin ZIF‐67 membranes using β‐Co(OH)_2_ nanosheets. a) The synthesis of β‐Co(OH)_2_ nanosheets; b) The process of converting β‐Co(OH)_2_ nanosheet coating to ZIF‐67 membrane.

## Synthesis of β‐Co(OH)_2_

Co(OH)_2_ is a layered transition metal hydroxide that has been extensively studied for application in energy storage^[^ ^21^, ^22^, ^23^, ^24^ ^]^ and electrocatalysis.^[^ ^25^, ^26^, ^27^, ^28^, ^29^ ^]^ We aimed to synthesize well‐dispersed ultrathin Co(OH)_2_ nanosheets, by a facile and scalable method as a self‐sacrificial template for ZIF‐67 membranes. Co(OH)_2_ nanosheets have been reported by either exfoliation (top‐down approach)^[^ ^25^, ^28^, ^30^ ^]^ or direct synthesis (bottom‐up approach).^[^ ^24^, ^29^, ^31^ ^]^ The top‐down approach requires several steps and usually suffers from low exfoliation efficiency. In contrast, the bottom‐up approach results in a high yield of nanosheets; however, the protocols either yield agglomerated sheets or require lengthy synthesis time.

One‐pot synthesis of ultrathin Co(OH)_2_ nanosheets has been reported previously. Room temperature synthesis of α‐Co(OH)_2_ nanosheets using cobalt salt and HmIm was reported in water‐methanol‐based mixed solvent.^[^ ^29^, ^31^ ^]^ However, the synthesized nanosheets were poorly dispersed and were agglomerated. This makes it challenging to make use of this suspension to fabricate thin films. Well‐dispersed atomic thin (∼ 0.5‐nm‐thick) β‐Co(OH)_2_ nanosheets using CoCl_2_ and aminoethanol in water have been reported.^[^ ^24^ ^]^ However, the synthesis protocol involved purging the reaction in nitrogen for several hours. The synthesis required several days, making it challenging to use for membrane application.

**Figure 1:**
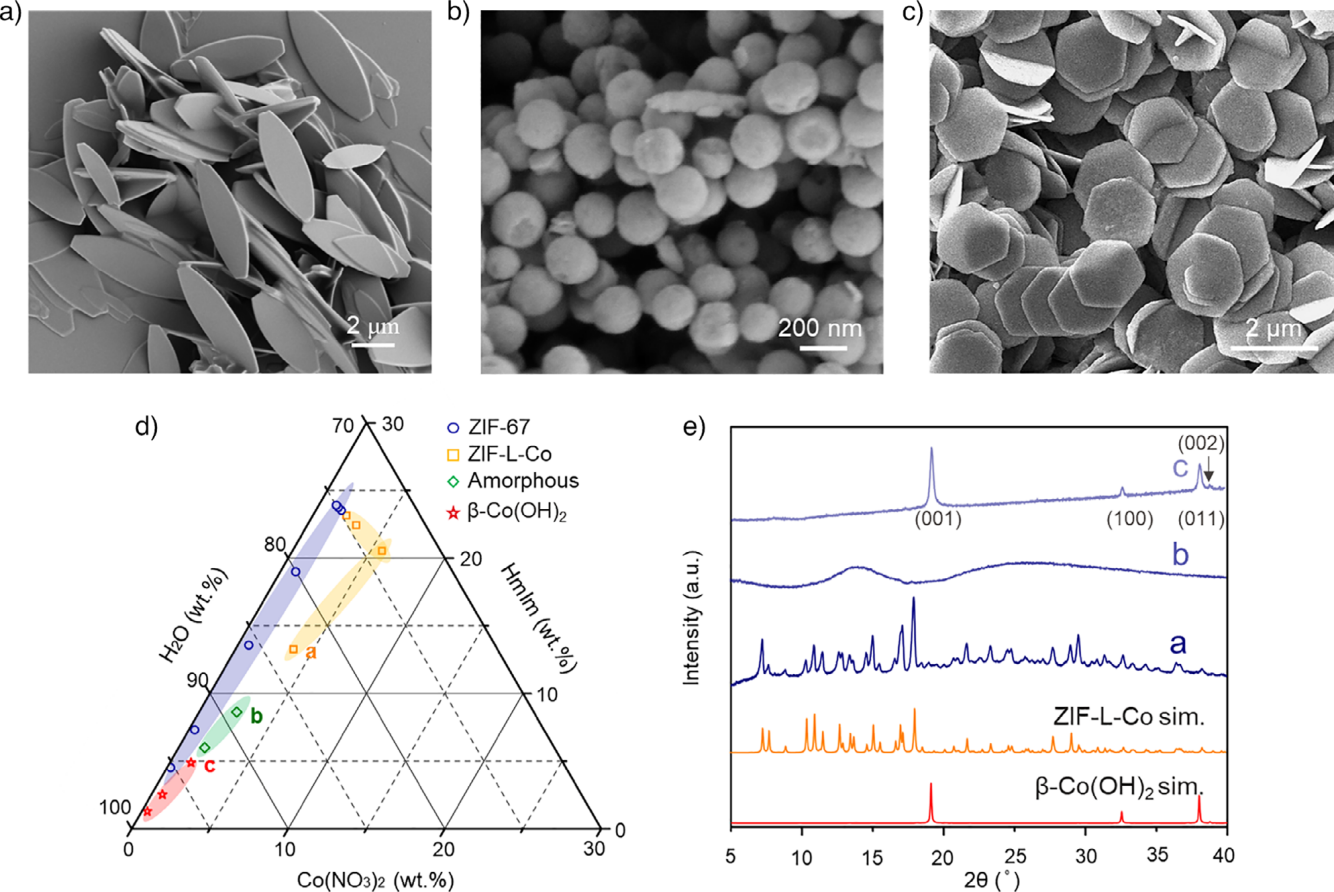
Characterization of materials synthesized using various Co/HmIm concentrations. a) Scanning electron microscopy (SEM) image of ZIF‐L‐Co synthesized using Co/HmIm concentration of 25 mM/200 mM; b) SEM image of amorphous spheres synthesized using Co/HmIm concentration of 15 mM/120 mM; c) SEM image of β‐Co(OH)_2_ platelets synthesized using Co/HmIm concentration of 8 mM/64 mM; d) A ternary phase diagram of the Co(NO_3_)_2_‐HmIm‐water system revealing precursor composition regimes that lead to ZIF‐67, ZIF‐L‐Co, amorphous material, and β‐Co(OH)_2_ platelets. e) Powder X‐ray diffraction (XRD) patterns of the materials shown in panels (a–c).

Herein, we report that ultradilute aqueous solutions of Co(NO_3_)_2_ and HmIm in water could yield ultrathin (∼1 nm) and well‐dispersed β‐Co(OH)_2_ nanosheets, at room temperature in just 1 h. This saves significant time and simplifies the synthesis of β‐Co(OH)_2_ as no purge environment was required. Our discovery emerged from attempts to use ultradilute precursor solutions to synthesize ZIF‐L‐Co. Typically, ZIF‐L‐Co synthesis follows the ZIF‐L‐Zn recipe,^[^ ^32^ ^]^ using a Co to HmIm concentration ratio of 25 mM/200 mM. For this concentration, we could reproduce the well‐established leaf‐like structure (Figure 1a) of ZIF‐L‐Co (see powder X‐ray diffraction (XRD) in Figure 1e). Next, we attempted synthesis using more diluted precursor solutions. Reducing the Co/HmIm concentrations to 15 mM/120 mM or 10 mM/80 mM produced spherical particles (Figures 1b and S1), which were identified as amorphous spheres (Figure 1e). At further dilution (8 mM/64 mM and 4 mM/32 mM), we obtained hexagonal platelets (Figures 1c and S1), confirmed as β‐Co(OH)_2_ by XRD (Figure 1e). Figure 1d illustrates a ternary phase diagram of the Co(NO_3_)_2_‐HmIm‐water system, revealing precursor composition regimes, which lead to ZIF‐67, ZIF‐L‐Co, amorphous material, and β‐Co(OH)_2_ platelets. These regimes form due to the state of HmIm in water solution at different concentrations. HmIm is a weak base and with its conjugate acid having a p*K*_a_ of ∼7.75,^[^ ^33^ ^]^ while its N–H group has a p*K*_a_ of ∼14.2.^[^ ^34^ ^]^ At low concentrations (lower pH, see Table S1), HmIm exists largely as 2‐methylimidazolium cations/neutral 2‐methylimidazole. OH^−^ ions in the solution coordinate with Co to form cobalt hydroxide. At moderate concentrations, 2‐methylimidazole starts to deprotonate to form 2‐methylimidazolate anions. Both OH^−^ ions and 2‐methylimidazolate ions would coordinate with Co, and the competition between the two coordination processes results in amorphous crystals lacking periodicity. At higher concentrations, 2‐methylimidazole is more prone to deprotonate to form 2‐methylimidazolate anions. The coordination of 2‐methylimidazolate ions with Co forms ZIF‐L‐Co crystals.

**Figure 2:**
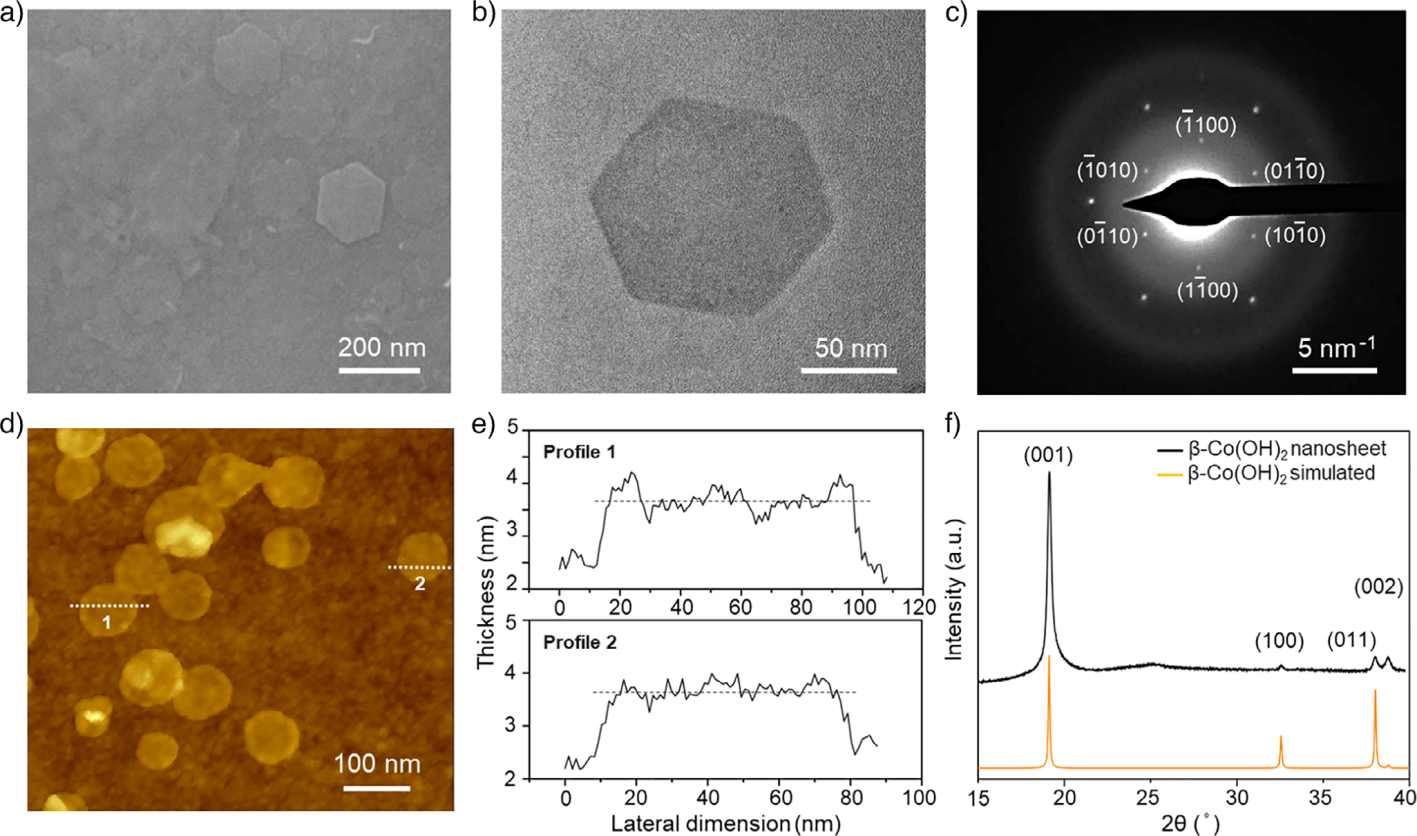
Characterization of β‐Co(OH)_2_ nanosheets synthesized using Co/HmIm concentration of 2 mM/16 mM. SEM a) and TEM b) images of the synthesized β‐Co(OH)_2_ nanosheets; c) Selected area electron diffraction (SAED) pattern of a β‐Co(OH)_2_ nanosheet. AFM image of β‐Co(OH)_2_ nanosheets deposited on Si wafer d) and the corresponding height profiles e) along the indicated white lines in (d). f) Powder XRD patterns of the synthesized β‐Co(OH)_2_ nanosheets.

Subsequent dilution of Co/HmIm to 2 mM/16 mM concentration resulted in predominantly thin β‐Co(OH)_2_ nanosheets (Figure 2a,b) instead of thick platelets at higher concentrations. This could be explained by the well‐established 2D nucleation and growth mechanism.^[^ ^35^ ^]^ At ultralow concentration, crystal growth follows 2D nucleation and growth (layer‐by‐layer growth mechanism).^[^ ^35^ ^]^ The rate‐limiting step for the layer‐by‐layer growth is the nucleation of a small 2D nucleus on the already formed 2D crystal surface. At ultradilute concentrations, the nucleation probability is much smaller compared to that at higher concentrations. This makes the 2D nanosheet crystals grown at low concentrations thinner than the ones grown at higher concentrations. This is likely as to why we obtained thinner and higher‐aspect‐ratio β‐Co(OH)_2_ nanosheets under ultradilute precursor condition (2 mM/16 mM Co/HmIm) compared to those obtained using higher precursor concentrations (4 mM/32 mM and 8 mM/64 mM Co/HmIm).

The synthesis of high‐aspect‐ratio β‐Co(OH)_2_ nanosheets could be achieved at room temperature for a synthesis time of just 1 h, by simply mixing the precursors in an open beaker. The nanosheets were well‐dispersed, as indicated by the discrete deposition of nanosheets on Si wafers as well as copper grids, the latter for transmission electron microscopy (TEM). The selected area electron diffraction (SAED) pattern (Figure 2c) exhibited a hexagonal lattice consistent with β‐Co(OH)_2_. Powder XRD further confirmed the β‐phase (Figure 2f). AFM analysis revealed the nanosheets to be extremely thin (∼1 nm, Figure 2d,e). This makes the aspect ratio (lateral size divided by thickness) of the sheet close to 100, which is attractive for achieving a dense coating of the sheets. Further, their excellent dispersion in water (as demonstrated by the Tyndall effect in Figure S2) makes it convenient for achieving a thin, compact coating on porous supports, which is key for producing high‐quality ZIF‐67 membranes.

## Preparing ZIF‐67 Membranes

**Figure 3:**
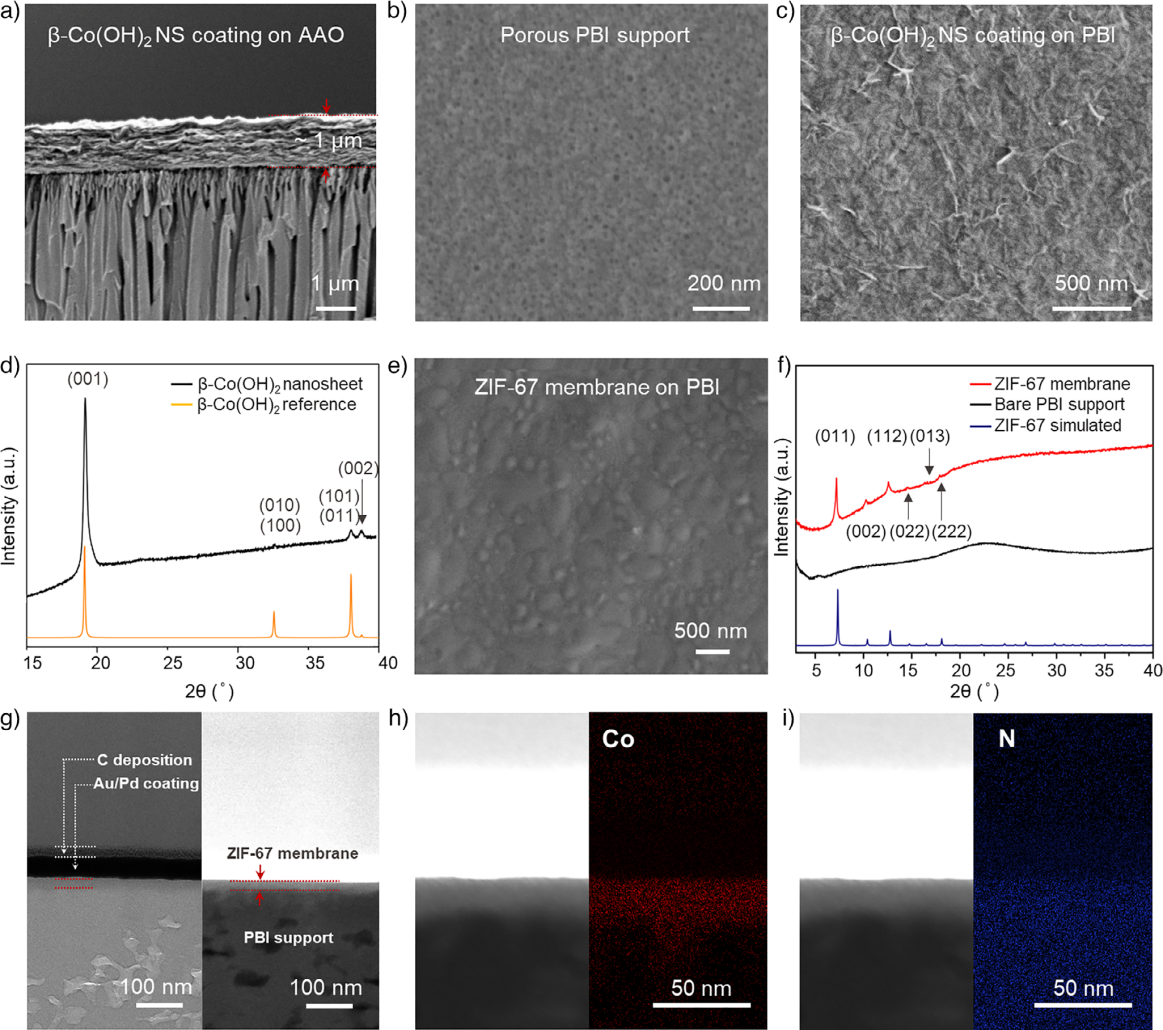
Characterization of the ZIF‐67 membrane made by converting β‐Co(OH)_2_ nanosheet coating. a) Cross‐sectional SEM image of the β‐Co(OH)_2_ nanosheet coating film prepared using 1 mL nanosheet dispersion on an AAO support; b) SEM image of the porous PBI support used to fabricate ZIF‐67 membranes; c) SEM image of the β‐Co(OH)_2_ nanosheet coating layer deposited on a PBI support; d) Out‐of‐plane XRD pattern of the β‐Co(OH)_2_ nanosheet coating layer on a PBI support demonstrating the preferred orientation in the c‐direction; e) SEM image of the prepared ZIF‐67 membrane; f) XRD pattern of the synthesized ZIF‐67 film as compared to ZIF‐67 simulated pattern and the bare PBI support pattern; g) Cross‐section HRTEM (left) and STEM (right) of the prepared ZIF‐67 membrane. Samples were prepared by FIB milling; h) and i) STEM‐EDX mapping of the cross section of the prepared ZIF‐67 membrane showing the element distribution of Co and N.

To prepare an ultrathin film of β‐Co(OH)_2_ as a precursor layer for ultrathin ZIF‐67, we optimized the coating process involving filtering a suspension of the β‐Co(OH)_2_ sheets on a porous support. For this, first, we evaluated the relationship between coating thickness and dispersion volume by filtering various amounts of nanosheet solution onto anodic aluminum oxide (AAO) supports. Use of 0.5 and 1.0 mL dispersions led to ∼0.5 and 1 µm thick films, respectively (Figures 3a and S3). This served as a calibration to adjust the amount of needed coating suspension for depositing an ultrathin β‐Co(OH)_2_ film. Accordingly, we used ∼40 µL dispersion to coat a homemade porous polybenzimidazole (PBI) support, hosting a pore opening diameter of ∼20 nm (Figure 3b) and yielding a high H_2_ permeance on the order of 10^−5^ mol m^−2^ s^−1^ Pa^−1^,^[^ ^36^ ^]^ with a diameter of 1 cm (Figure S4). The coating could be achieved in a short deposition time (∼2 min). This led to a smooth, continuous, compact, and oriented film, attributed to the high aspect ratio of the nanosheets (Figure 3c). The preferred orientation along the *c*‐out‐of‐plane direction was demonstrated by the out‐of‐plane XRD pattern of the β‐Co(OH)_2_ nanosheet coating film (Figure 3d), as evidenced by weak (100)/(010) peaks and strong (001) and (002) peaks. The cross‐section of the coating layer showing horizontal packing of the nanosheets (Figure 3a) also corroborated the highly preferential orientation of the obtained film.

The ultrathin and compact β‐Co(OH)_2_ nanosheet coatings serve as a promising candidate as a sacrificial precursor layer for conversion to ZIF‐67 membranes. For conversion, we carried out a thermal treatment of the film by exposing it to a dilute HmIm solution. During the conversion, HmIm would coordinate with Co, and β‐Co(OH)_2_ would be transformed to ZIF‐67. The transformation is reconstructive, and the dense β‐Co(OH)_2_ layer would be converted to porous ZIF‐67 membrane, enabling its gas sieving property. Briefly, ∼1 mL of 16 mM HmIm solution in water was placed on the β‐Co(OH)_2_ nanosheet film resting on PBI (Figure S5). Subsequently, the film was heat‐treated to 120 °C for 2 h. This thermal treatment successfully converted the β‐Co(OH)_2_ coating layer to ZIF‐67, as confirmed by XRD measurement (Figure 3f). The conversion of β‐Co(OH)_2_ to ZIF‐67 was complete, as evidenced from the lack of XRD peaks associated with β‐Co(OH)_2_. The SEM image (Figure 3e) showed that the converted ZIF‐67 grains were well intergrown, owing to the compact coating of the β‐Co(OH)_2_ precursor layer.

Cross‐sectional specimens for high‐resolution TEM (HRTEM) and scanning TEM (STEM) were prepared using focused‐ion beam (FIB). The corresponding HRTEM and STEM images demonstrated that the prepared ZIF‐67 membranes were ultrathin, with thickness of just ∼20 nm (Figure 3g). Energy‐dispersive X‐ray spectroscopy (EDX, Figures 3h,i and S6) was used to map the distribution of elements of the membrane. The presence of an ultrathin Co layer confirmed the presence of an ultrathin ZIF‐67 layer on the porous PBI support.

**Figure 4:**
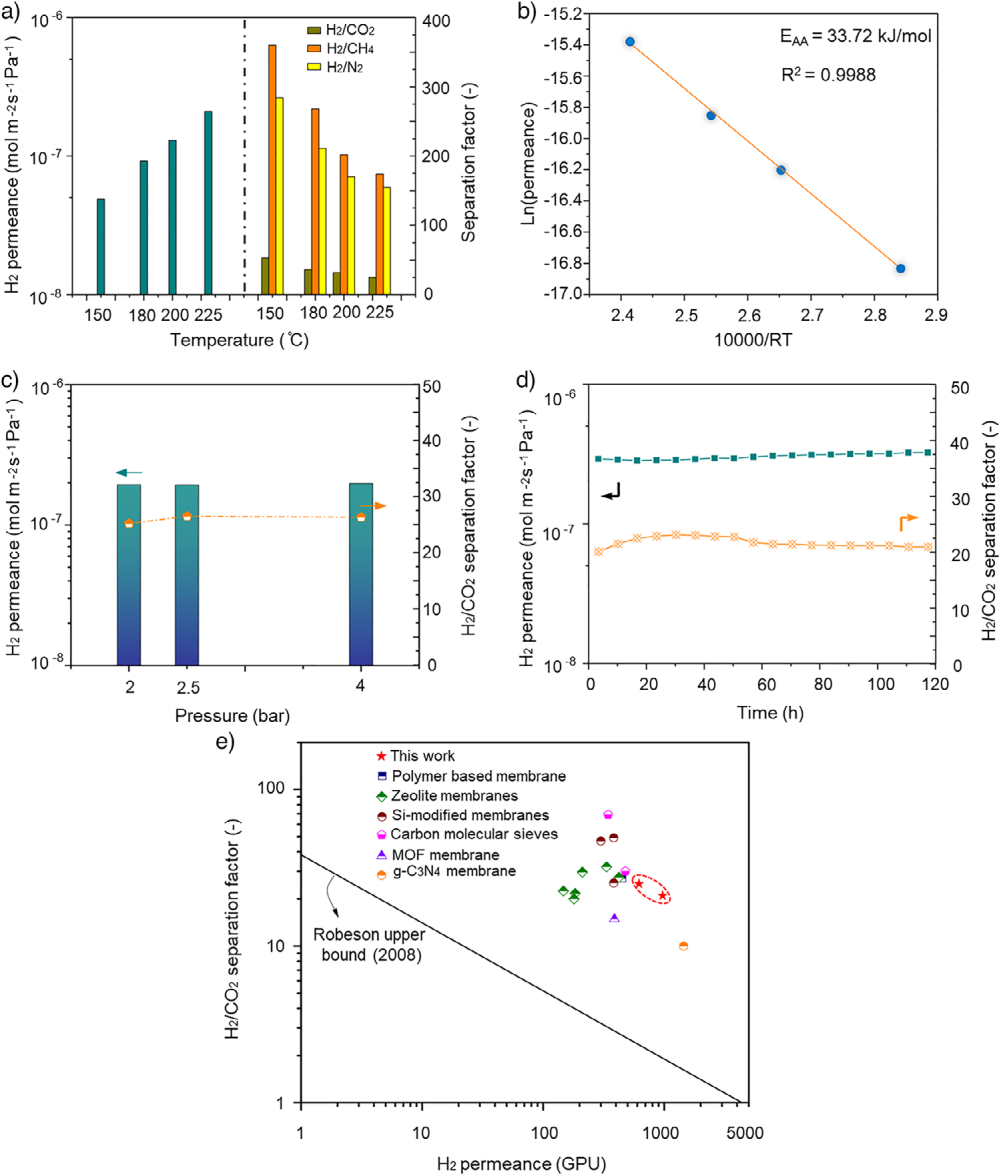
a) Gas permeation results showing H_2_ permeance as a function of testing temperature and the corresponding H_2_/CO_2_, H_2_/CH_4_, and H_2_/N_2_ separation factors; b) Arrhenius plot for hydrogen permeance as a function of temperature showing that hydrogen transport was in temperature‐activated regime; c) H_2_/CO_2_ separation performance tested at 225 °C with an equimolar H_2_/CO_2_ mixture feed at varying feed pressures; d) Membrane stability test using a 50/50 mol% H_2_/CO_2_ gas mixture with ∼1.5 mol% water vapor at 250 °C; e) Comparison with state‐of‐the‐art membranes for H_2_/CO_2_ separation at temperatures higher than 200 °C. The literature data corresponds to reported H_2_/CO_2_ performance data from pressurized feed conditions.

Gas permeation measurements were conducted to evaluate membrane performance. Before converting to ZIF‐67, the β‐Co(OH)_2_ coating layer yielded an H_2_ permeance of 2.6 × 10^−7^ mol m^−2^ s^−1^ Pa^−1^ at room temperature with H_2_/CO_2_, H_2_/CH_4_, and H_2_/N_2_ selectivities close to Knudsen selectivities (Table S2). Since β‐Co(OH)_2_ is a dense material, the gas transport should be attributed to pinhole defects in the β‐Co(OH)_2_ coating layer. After converting to ZIF‐67, these defects were eliminated, and the intrinsic porous structure of ZIF‐67 enabled the membrane with gas sieving properties. As shown in Figure 4a, the membrane exhibited temperature‐activated transport, evidenced by the increase of H_2_ permeance as an exponential function of the temperature. H_2_ permeance increased from 4.9 × 10^−8^ mol m^−2^ s^−1^ Pa^−1^ at 150 °C to 9.2 × 10^−8^ mol m^−2^ s^−1^ Pa^−1^ at 180 °C. It further increased to 1.3 × 10^−7^ and 2.1 × 10^−7^ mol m^−2^ s^−1^ Pa^−1^ at 200 and 225 °C, respectively. The apparent activation energy (*E*_AA_) for H_2_ transport was calculated by fitting the permeation data with the Arrhenius equation (Figure 4b) and was estimated to be 33.7 kJ mol^−1^. The presence of this energy barrier confirms confined transport of H_2_ from small pores in ZIF‐67 resulting in molecular sieving. The corresponding H_2_ separation factors over CO_2_, CH_4_, and N_2_ were ∼50, 280, and 360 at 150 °C, slightly decreasing with temperature to ∼25, 150, and 180, respectively, at 225 °C. The high separation performance at high temperatures makes the ultrathin ZIF‐67 membrane highly attractive for H_2_/CO_2_, H_2_/CH_4_, and H_2_/N_2_ separations, relevant in applications related to precombustion carbon capture, off‐gas hydrogen recovery, and hydrogen separation in ammonia production, etc.

Membrane stability under humid conditions was also evaluated. The membrane maintained stable performance over 120 h when a 50/50 mol% H_2_/CO_2_ mixture with ∼1.5 mol% water vapor was fed at 250 °C (Figure 4d). It yielded an attractive H_2_ permeance (∼3.0 × 10^−7^ mol m^−2^ s^−1^ pa^−1^) and H_2_/CO_2_ selectivity > 20, demonstrating resistance to high‐temperature humid feed. The effect of feed pressure was also studied for H_2_/CO_2_ separation. Figure 4c demonstrated the separation performance of a prepared ZIF‐67 membrane tested at 225 °C with an equimolar H_2_/CO_2_ mixture feed at varying feed pressures. The membrane separation performance was not affected by higher feed pressures (tested up to 4 bar), which indicates that the membrane is stable against pressurization. It should be noted that stable performance under feed pressurization is crucial for practical application of membranes. Figure 4e summarizes the state‐of‐the‐art membranes for H_2_/CO_2_ separation at temperatures higher than 200 °C, and where membranes were evaluated in pressurized feed conditions (Table S3). The ultrathin ZIF‐67 membranes compared favorably with the literature where pressurized membrane data is reported. These results underscore the membrane's attractive H_2_ sieving performance at high temperature, suitable for applications for hydrogen purification, including pre‐combustion carbon capture.^[^ ^37^ ^]^

In conclusion, we have demonstrated a simple and scalable method for fabricating ultrathin well‐intergrown ZIF‐67 membranes using ∼1 nm‐thick β‐Co(OH)_2_ nanosheets as a self‐sacrificial coating. The membranes, with a thickness of ∼20 nm on porous polymeric support, exhibit attractive and stable hydrogen permeance and H_2_/CO_2_ selectivity. These properties make these membranes highly attractive candidates for high‐temperature gas separation applications, particularly in precombustion carbon capture. The simplicity of this method opens new avenues for extending sacrificial nanosheet coatings to fabricate other MOF membranes based on cobalt. This also opens the path to tune the chemistry to customize membrane performance for diverse separations.

## Supporting Information

The authors have cited additional references within the Supporting Information.^[^ ^36^, ^37^, ^38^, ^39^, ^40^, ^41^, ^42^, ^43^, ^44^, ^45^, ^46^, ^47^, ^48^, ^49^, ^50^, ^51^, ^52^, ^53^, ^54^, ^55^ ^]^

## Conflict of Interests

The authors declare no conflict of interest.

## Supporting information



Supporting Information

## Data Availability

The data that support the findings of this study are available from the corresponding author upon reasonable request.
