# Supplementary material for: Facile Synthesis of 20‐nm‐Thick ZIF‐67 Films for Hydrogen Sieving Using β‐Co(OH)2 Precursor Nanosheets
Source: Angew Chem Int Ed Engl. 2025 Sep 21;64(45):e202516048. doi: 10.1002/anie.202516048 (PMC12582003; doi:10.1002/anie.202516048)
Supplement: Supplementary file 1 — Supporting Information [file ANIE-64-e202516048-s001.docx]

Supporting Information

**Facile Synthesis of 20-nm-Thick ZIF-67 Films for Hydrogen Sieving using β-Co(OH)₂ Precursor Nanosheets**

Xuekui Duan,^[a]^ Yueqing Shen,^[a]^ and Kumar Varoon Agrawal*^[a]^

**Materials**

Cobalt nitrate hexahydrate (CNH) (Co(NO_3_)_2_.6H_2_O, 98%) and 2-Methylimidazole (HmIm, C_4_H_6_N_2_, 99%) were purchased from Sigma-Aldrich. PBI-AM Fumion® powder was obtained from Fumatech. Stainless-steel mesh (pore size 20 μm, Part number #325X2300TL0014) was obtained from TWP Inc.

**Synthesis of Co(OH)_2_ Nanosheets**

The synthesis of Co(OH)_2_ nanosheets was achieved using ultra-dilute 2-methylimidazole aqueous solution as the base. Briefly, a Co ion solution was prepared by dissolving 0.0582 g Co(NO_3_)_2_.6H_2_O in 90 mL ultrapure water (18.2 MΩ·cm). In parallel, an HmIm solution was prepared by dissolving 0.1314 g HmIm in 10 mL ultrapure water (18.2 MΩ·cm). After mixing well separately, the Co ion solution was added to the HmIm solution at once. The mixture was kept stirring at room temperature for 1 hour. The final molar concentrations of the reactants were CNH : HmIm = 2 mM : 16 mM. After the completion of reaction, samples were taken from the reaction mixture, and drop-coated on silicon wafer/copper grid for scanning electron microscopy (SEM), atomic force microscopy (AFM) and transmission electron microscopy (TEM) analysis. Since the as-synthesized solution contains both thin and thicker nanosheets, a centrifugation step (15000 rpm for 10 min) was carried out to remove thicker nanosheets. After centrifugation, the supernatant contains mostly ultra-thin Co(OH)_2_ nanosheets (~ 1 nm). This nanosheet dispersion was used for further coating on porous substrate.

**Membrane Preparation**

Porous PBI-AM supports were used for the preparation of sub-20 nm ZIF-67 membranes. These supports were prepared by the non-solvent induced phase inversion method as reported in literature. [36] Briefly, a polymer dope solution having concentration of 8% (w/w) was prepared by adding commercial PBI-AM Fumion® powder in 1-Methyl-2-pyrrolidinone (NMP) and mixing vigorously using mechanical stir. After dissolving the PBI powder in NMP, the solution was centrifuged at 40,000 g-force for 3 hours to settle down any undissolved particles. PBI-AM films were casted on stainless-steel metal mesh using a doctor blade casting system. The as-cast films were coagulated in a 60 °C deionized (DI) water bath for 9 hours to allow complete phase inversion and then washed thoroughly with deionized (DI) water before drying. Finally, the supports were dried at room temperature followed by heat treatment at 330 °C for 8 hours with a heating and cooling rate of 0.5 °C /min.

The prepared PBI-AM support was sealed in a home-made filtration cell with Viton® gaskets (Figure S4). The design of the filtration cell was reported in our early report. [38] The assembled filtration cell was placed on a vacuum filtration set-up. Then, ~ 40 µL of Co(OH)_2_ nanosheet dispersion were dropped onto the PBI support. After ensuring the support be completely wetted, vacuum was started and the back side of PBI support was maintained under vacuum. Under the vacuum driving force, nanosheets would deposit horizontally on the PBI surface, generating a thin and compact coating layer. After coating, ~ 1 mL of 16 mM HmIm aqueous solution was added in the home-made filtration cell. The cell was placed in a 120 °C oven for 2 hours. The Co(OH)_2_ coating was converted to ZIF-67 membranes under this simple heat treatment.

**Characterization**

Scanning electron microscopy (SEM) images were collected using an FEI Teneo scanning electron microscope with Schottky Field Emission Gun at an acceleration voltage of 1-2 kV and in-lens detector. Samples were sputter-coated with ~ 5 nm thick Iridium layer before imaging to minimize sample charging effect. Transmission electron microscopy (TEM) images were collected using an FEI Tecnai G2 Spirit transmission electron microscope with a LaB6 source at an acceleration voltage of 120 kV. Focused-ion beam milling was performed using an FIB-SEM Zeiss CrossBeam 540. High resolution TEM and STEM images were obtained using Talos F200S transmission electron microscope which has equipped with precise EDX (energy dispersive X-ray spectroscopy) analysis. X-ray diffraction (XRD) patterns were recorded using a Bruker D8 Discover with CuKα radiation (λ= 1.5406 Å).

**Gas permeation**

Mixed gas permeation tests were performed to evaluate the separation performance of the produced ZIF-67 membranes. The tests were carried out using a homemade permeation setup as reported earlier. [38] Briefly, 60-120 mL/min 50/50 mol% gas mixture (H_2_/CO_2_, H_2_/N_2_, or H_2_/CH_4_) was fed to the membrane through the feed inlet. The pressure of feed was adjusted between 2 to 4 bars and maintained constant. At the permeate side, Ar was used as the sweep gas and the pressure was kept at 1 bar. The permeation cell was placed in an oven which was used to control the temperature of permeation tests. During test, the chemical potential difference would drive part of the feed gas through the membrane. The permeated gas would be removed by the sweep gas and sent to a mass spectrometer (Hiden Analytical, HPR-20) for a real time analysis. The steady state data was used to calculate the gas concentrations in permeate and further determine the gas permeances and membrane separation factors.


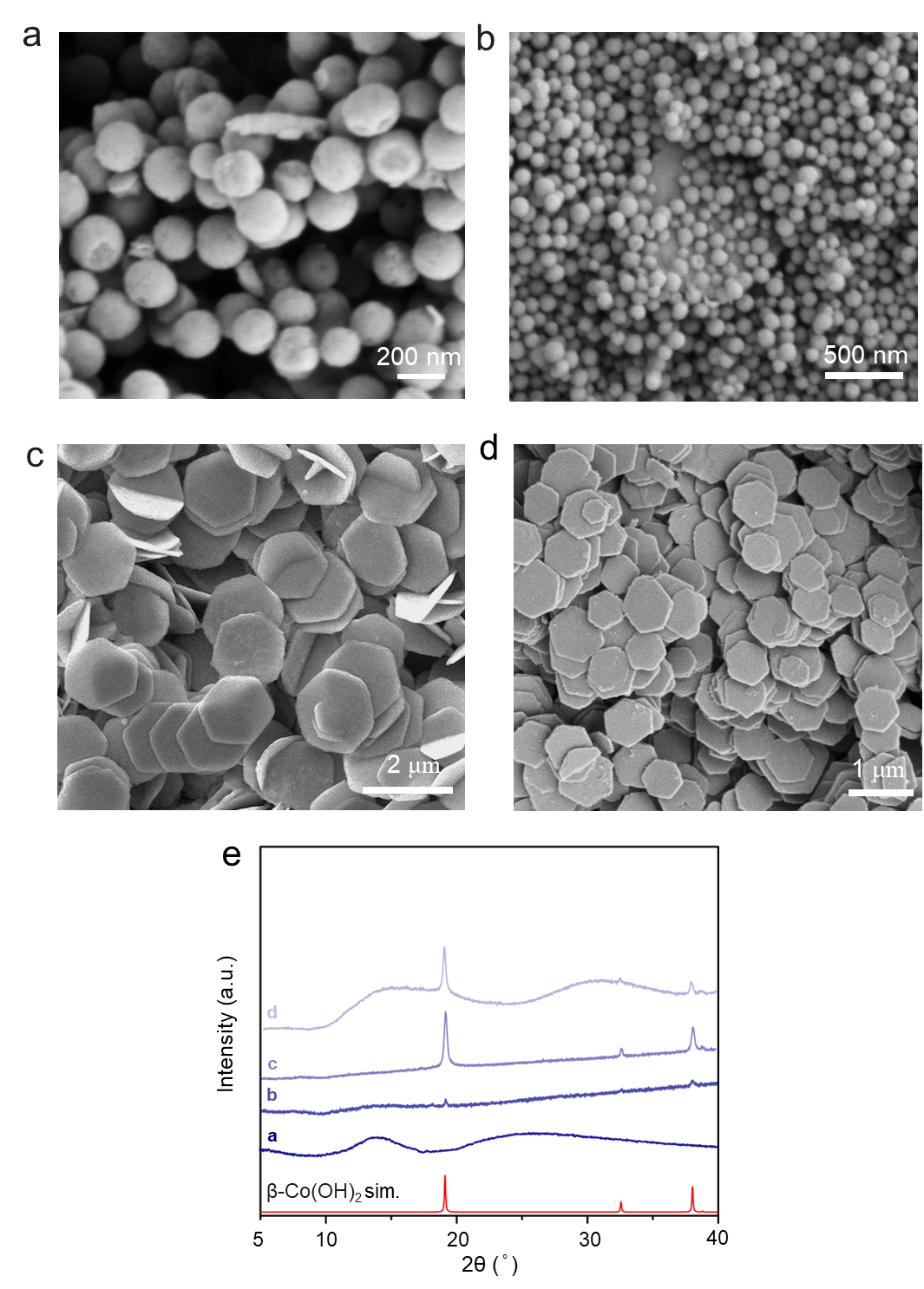


**Figure S1**. More characterization of materials synthesized using various Co/HmIm concentrations. a) SEM image of amorphous spheres synthesized using Co/HmIm concentration of 15 mM/120 mM; b) SEM image of amorphous spheres synthesized using Co/HmIm concentration of 10 mM/80 mM, with occasional hexagonal platelets which were identified β-Co(OH)_2_ platelets; c) SEM image of β-Co(OH)_2_ platelets synthesized using Co/HmIm concentration of 8 mM/64 mM; d) SEM image of β-Co(OH)_2_ nanoplatelets synthesized using Co/HmIm concentration ratio of 4 mM/32 mM; e) Powder X-ray diffraction (XRD) patterns of the materials shown in a)-d).


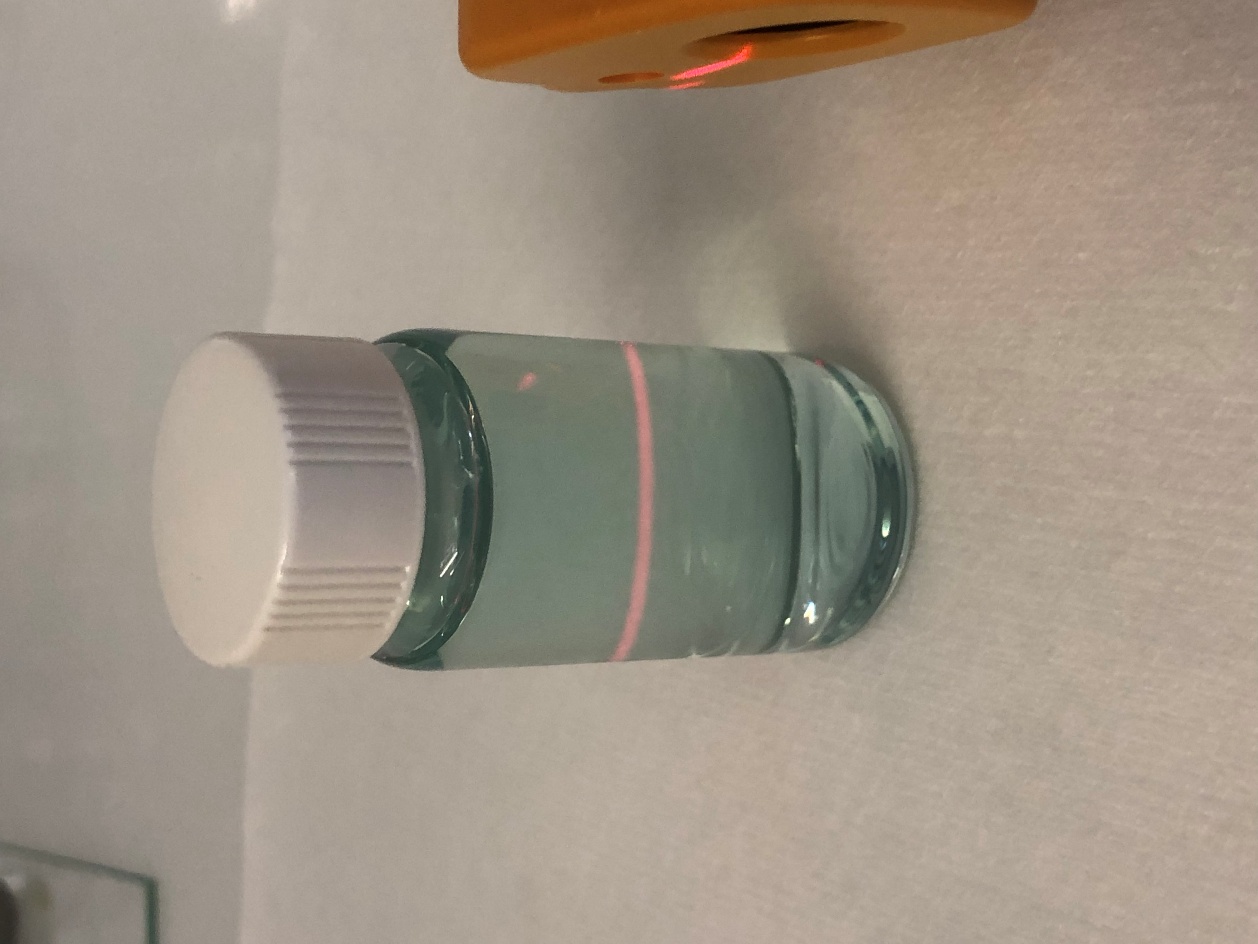


**Figure S2.** Tyndall effect of the as-synthesized the β-Co(OH)_2_ nanosheet in water, demonstrating the excellent dispersion of the nanosheets in water solution.


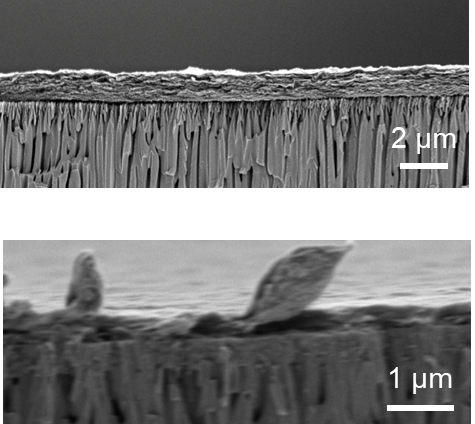


**Figure S3.** Cross-sectional SEM image of the films prepared using 1 mL (top) or 0.5 mL (bottom) nanosheet dispersion on AAO supports.


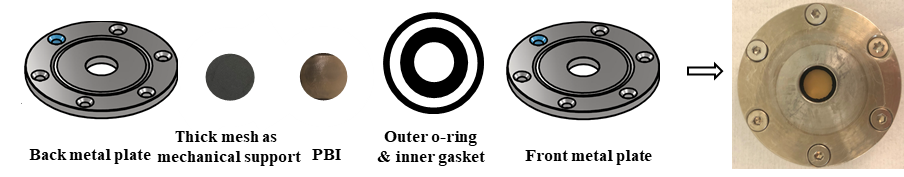


**Figure S4.** Assembly of the home-made filtration module.[38]


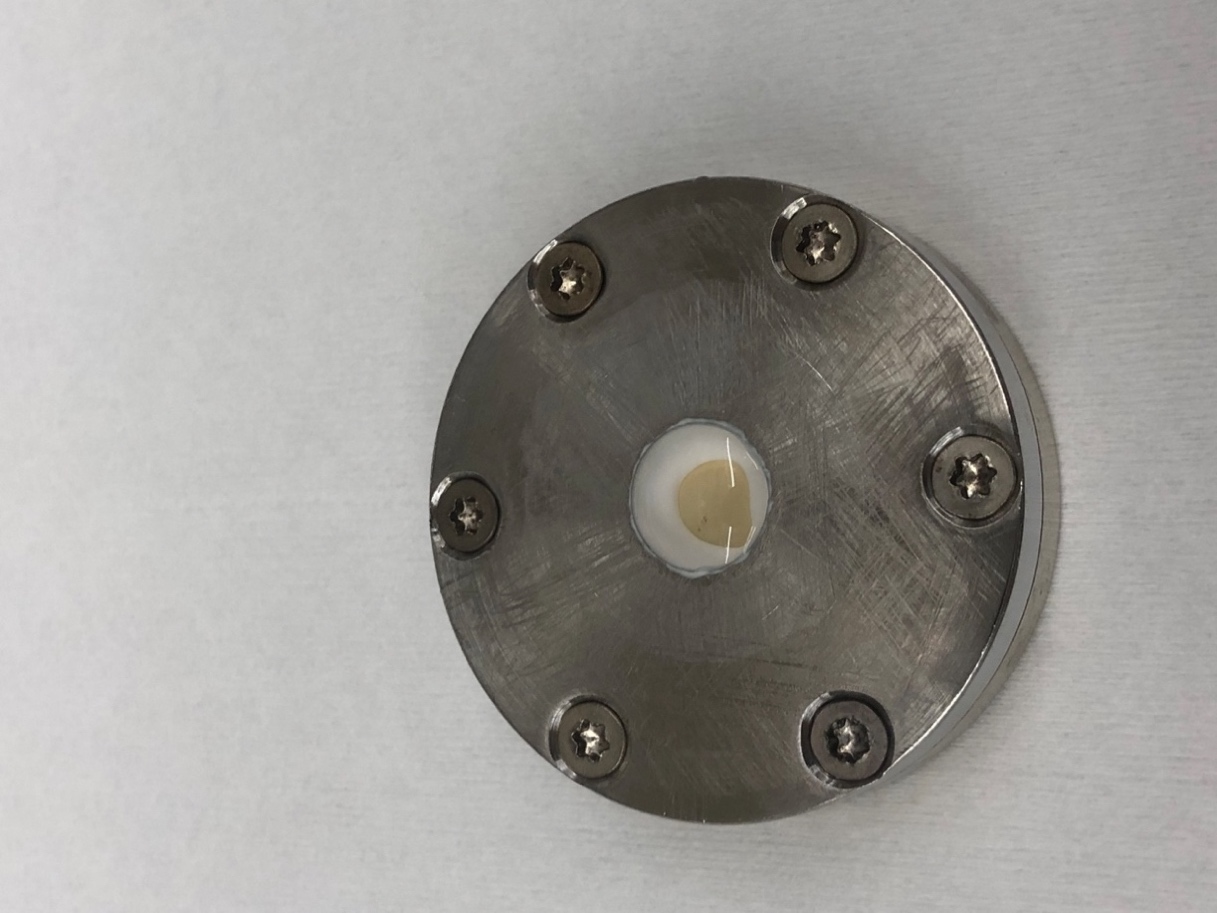


**Figure S5.** Illustration of the process for membrane fabrication by adding ~ 1 mL of 16 mM HmIm solution in water onto the β-Co(OH)_2_ nanosheet film coated on PBI in the membrane module. The white part is epoxy glue used to seal the o-ring and membrane interface.


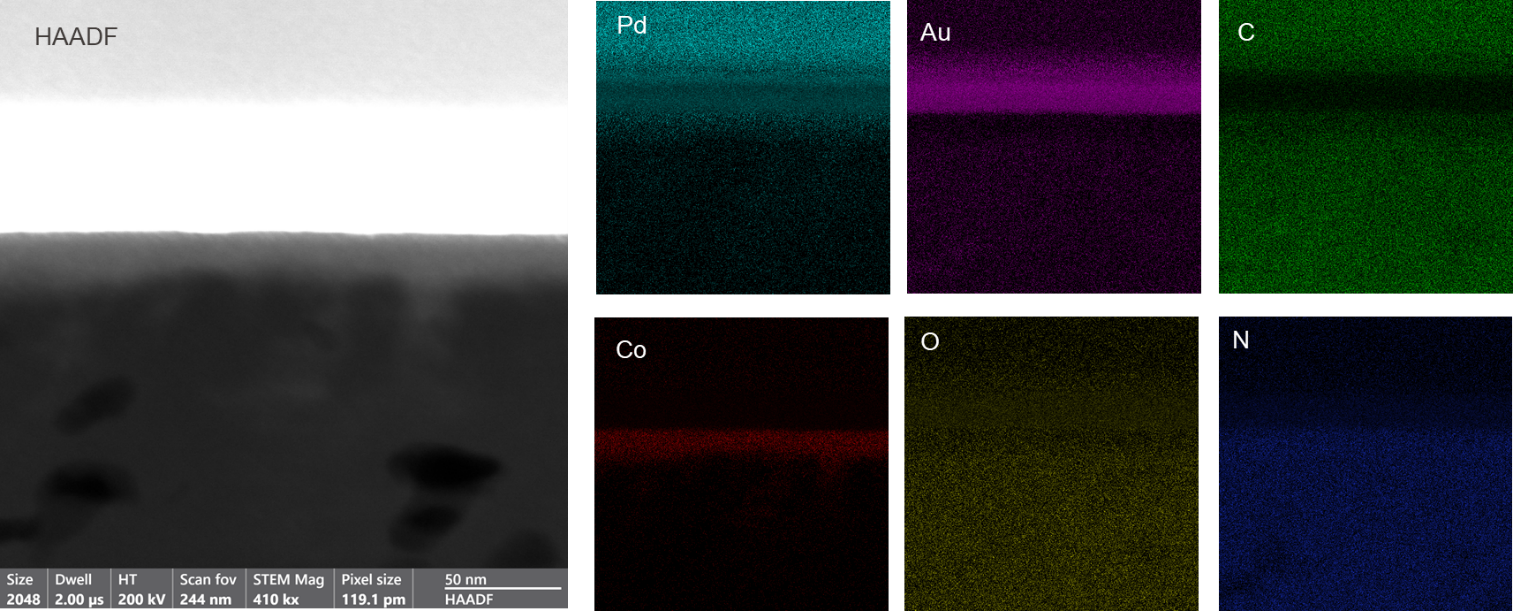


**Figure S6.** STEM-EDX mapping of the cross section of the prepared ZIF-67 membrane showing the element distribution of Pd, Au, C, Co, O and N. Pd and Au originated from the Pd/Au sputtering coating used to protect the ZIF-67 layer before FIB milling.

**Table S1.** pH of aqueous HmIm solution at various concentrations.

| HmIm concentration | 16 mM | 32 mM | 64 mM | 80 mM | 120 mM | 200 mM |
| --- | --- | --- | --- | --- | --- | --- |
| pH | 10.1 | 10.3 | 10.4 | 10.5 | 10.6 | 10.7 |

**Table S2.** Permeation data of β-Co(OH)_2_ film on PBI support before conversion to ZIF-67.

| Membrane | Feed pressure | T (°C) | Gas permeance (mol m^-2^ s^-1^ Pa^-1^) | | | |
| --- | --- | --- | --- | --- | --- | --- |
|  |  |  | H_2_ | CO_2_ | CH_4_ | N_2_ |
| β-Co(OH)_2_ film  on PBI | 1.8 bar | ~25 | 2.6 × 10^-7^ | 5.0 × 10^-8^ | 6.9 × 10^-8^ | 6.2 × 10^-8^ |

**Table S3.** Literature data of the H_2_/CO_2_ separation performance from the state-of-the-art membranes at temperatures higher than 200 °C.

| Membrane | Feed | Pressure | T (°C) | H_2_ (GPU) | H_2_/CO_2_ | Ref. |
| --- | --- | --- | --- | --- | --- | --- |
| LANL PBI HFM | Mixture | - | 250 | 500 | 19 | [39] |
| T_300_-C-BILP | Mixture | 0 barg | 250 | 493 | 28.5 | [40] |
|  | Mixture | 0 barg | 300 | 215 | 40 |  |
| PBI/ZIF-8 | Pure | 2.5 barg | 230 | 450 | 27 | [41] |
| MFI (pure phase) | Mixture | - | 450 | 1665 | 10 | [42] |
| B-ZSM-5 silylated | 50:50 | 1.38 barg | 400 | 300 | 47 | [43] |
| Modified MFI (with silica) | Pure | - | 450 | 1185 | 141 | [44] |
| MFI bilayer (with silica) | 50:50 | 1 barg | 450 | 383 | 25.3 | [45] |
| DDR | Pure |  | 500 | 83.5 | 7.6 | [46] |
| RUB-15 on AAO | 50:50 | 1 barg | 250 | 211 | 29.7 | [47] |
|  |  |  |  | 184 | 21.7 |  |
|  |  |  | 300 | 424 | 27.5 |  |
|  |  |  |  | 147 | 22.5 |  |
|  |  |  |  | 334 | 32.1 |  |
| ZIF 7 | 50:50 | 0 barg | 220 | 135 | 13.6 | [48] |
| ZIF 7 | 50:50 | 0 barg | 300 | 1077 | 7.2 | [49] |
| ZIF 7 | 50:50 | 0 barg | 350 | 1856 | 6.0 | [50] |
| ZIF 95 | 50:50 | 0 barg | 325 | 5795 | 25.7 | [51] |
| ZnO converted ZIF | - | 0 barg | 250 | 602 | 53 | [52] |
| ZIF/GO | 50:50 | 1 barg | 250 | 388 | 14.9 | [53] |
| g-C_3_N_4_ | Pure | 2 barg | 250 | 1450 | 10 | [54] |
| RUB-15 on PBI support | 50:50 | 1-3.3 barg | 250 | 180 | 20 | [38] |
| Hybrid silica | mixture | 5 barg | 250 | 383 | 49.2 | [55] |
| Novolac resin-based CMS | mixture | 12.8 barg | 250 | 478 | 30 | [56] |
| Catalyst packed CMS | mixture | 24 barg | 250 | 344 | 69 | [57] |
| Ultrathin ZIF-67 | mixture | 1-3 barg | 225 | 620 | 25 | This work |
|  | mixture | 1 barg | 250 | 970 | 21 |  |
